# Supplementary material for: Cavin1 intrinsically disordered domains are essential for fuzzy electrostatic interactions and caveola formation
Source: Nat Commun. 2021 Feb 10;12:931. doi: 10.1038/s41467-021-21035-4 (PMC7875971; doi:10.1038/s41467-021-21035-4)
Supplement: Supplementary file 1 — Supplementary Information [file 41467_2021_21035_MOESM1_ESM.pdf]

SUPPLEMENTARY INFORMATION

Cavin1 intrinsically disordered domains are essential for fuzzy electrostatic interactions and caveola formation

Vikas A. Tillu[1], James Rae[1,2], Ya Gao[1], Nicholas Ariotti[3,4], Matthias Floetenmeyer[2], Oleksiy Kovtun[1,5], Kerrie-Ann McMahon[1], Natasha Chaudhary[1,6], Robert G. Parton[1,2]* and Brett M. Collins[1]*

Supplementary References

1. Oates, M.E. *et al.* D2P2: database of disordered protein predictions. *Nucleic Acids Research* **41**, D508-D516 (2012).

2. Simossis, V.A. & Heringa, J. PRALINE: a multiple sequence alignment toolbox that integrates homology-extended and secondary structure information. *Nucleic Acids Res* **33**, W289-294 (2005).

Supplementary Table 1. Primers used in this study.

Number	List of Primers used in the study		
1	V462_pHUE_mC1F	tggtgttgcgcctccgcGGTGGA ATGGAGGATGTCACGCTC	To clone Cavin1 truncation mutants in pHUE vector
2	V465_pHUE_mC1_45F	tggtgttgcgcctccgcGGTGGA CTGATCAAGTCGGAC	
3	V466_pHUE_mC1_R	ctcgaattcggatccaccgcTTA GTCGCTGTCGCTCTT	
4	V467_pHUE_mC1_310R	ctcgaattcggatccaccgcTTA ATACACCACATGGTC	
5	V468_pHUE_mC1_345R	ctcgaattcggatccaccgcTTA CTCCACCATCTCGGT	
6	V480_pOPIN_HisUB_F	CACAGCAGCGGTGGATCC CATCATCACAGCAGCGGC	To clone His-UB-Cavin1 full length and truncates in pOPINGFP vector
7	V481_pOPIN_mC1_R	CCCTTGCTCACGGATCC GTCGCTGTCGCTCTT	
8	V482_pOPIN_mC1_310R	CCCTTGCTCACGGATCC ATACACCACATGGTC	
9	V485_mC1_HUE_345_M1F	gtgttgcgcctccgcGGTGGA ATGGCTGCCGTCACG	To clone cavin1 (1-345) mutants in pHUE and eGFP vector
10	V486_mC1_HUE_345_M1R	gaattcggatccaccgcTTA CTCCACCATCTCGGT	
11	V487_mC1_HUE_345_M2F	gtgttgcgcctccgcGGTGGA ATGGAGGATGGCGGC	
12	V488_mC1_HUE_345_M2R	gaattcggatccaccgcTTA CTCCACCATCTCGGT	
13	V489_mC1_HUE_345_M3F	gtgttgcgcctccgcGGTGGA ATGGAGGATGTCACG	
14	V490_mC1_HUE_345_M3R	gaattcggatccaccgcTTA GGCCACCATGGCGGT	
15	V491_mC1_HUE_345_M4F	gtgttgcgcctccgcGGTGGA ATGGAGGATGTCACG	
16	V492_mC1_HUE_345_M4R	gaattcggatccaccgcTTA CTCGCCGCTCTCGCC	
17	V493_mC1_HUE_345_M5F	gtgttgcgcctccgcGGTGGA ATGGAGGATGTCACG	
18	V494_mC1_HUE_345_M5R	gaattcggatccaccgcTTA CTCCACCATCTCGGT	
19	V495_mC1_eGFP_345_M1F	TCGAGCTCAAGCTTCGAATTCT ATGGCTGCCGTCACG	

20	V496_mC1_eGFP_345_M1R	CGTCGACTGCAGAATTTTA CTCCACCATCTCGGT	
21	V497_mC1_eGFP_345_M2F	TCGAGCTCAAGCTTCGAATTCT ATGGAGGATGGCGGC	
22	V498_mC1_eGFP_345_M2R	CGTCGACTGCAGAATTTTA CTCCACCATCTCGGT	
23	V499_mC1_eGFP_345_M3F	TCGAGCTCAAGCTTCGAATTCT ATGGAGGATGTCACG	
24	V500_mC1_eGFP_345_M3R	CGTCGACTGCAGAATTTTA GGCCACCATGGCGGT	
25	V501_mC1_eGFP_345_M4F	TCGAGCTCAAGCTTCGAATTCT ATGGAGGATGTCACG	
26	V502_mC1_eGFP_345_M4R	CGTCGACTGCAGAATTTTA CTCGCCGCTCTCGCC	
27	V503_mC1_eGFP_345_M5F	TCGAGCTCAAGCTTCGAATTCT ATGGAGGATGTCACG	
28	V504_mC1_eGFP_345_M5R	CGTCGACTGCAGAATTTTA CTCCACCATCTCGGT	
29	V510_mCherryC1_mC1_F	TCTCGAGCTCAAGCTTCGAATTCT ATGGAGGATGTCACGCTCC	To clone cavin1 (1-345) mutants in pHUE and eGFP vector
30	V511_mCherryC1_mC1_R	GGTACCGTCGACTGCAGAATT TTA GTCGCTGTCGCTCTTGTC	
31	V512_mCherryC1_mC1_45F	TCTCGAGCTCAAGCTTCGAATTCT ATGCTGATCAAGTCGGAC	
32	V513_mCherryC1_mC1_310R	GGTACCGTCGACTGCAGAATT TTA ATACACCACATGGTCGGGC	

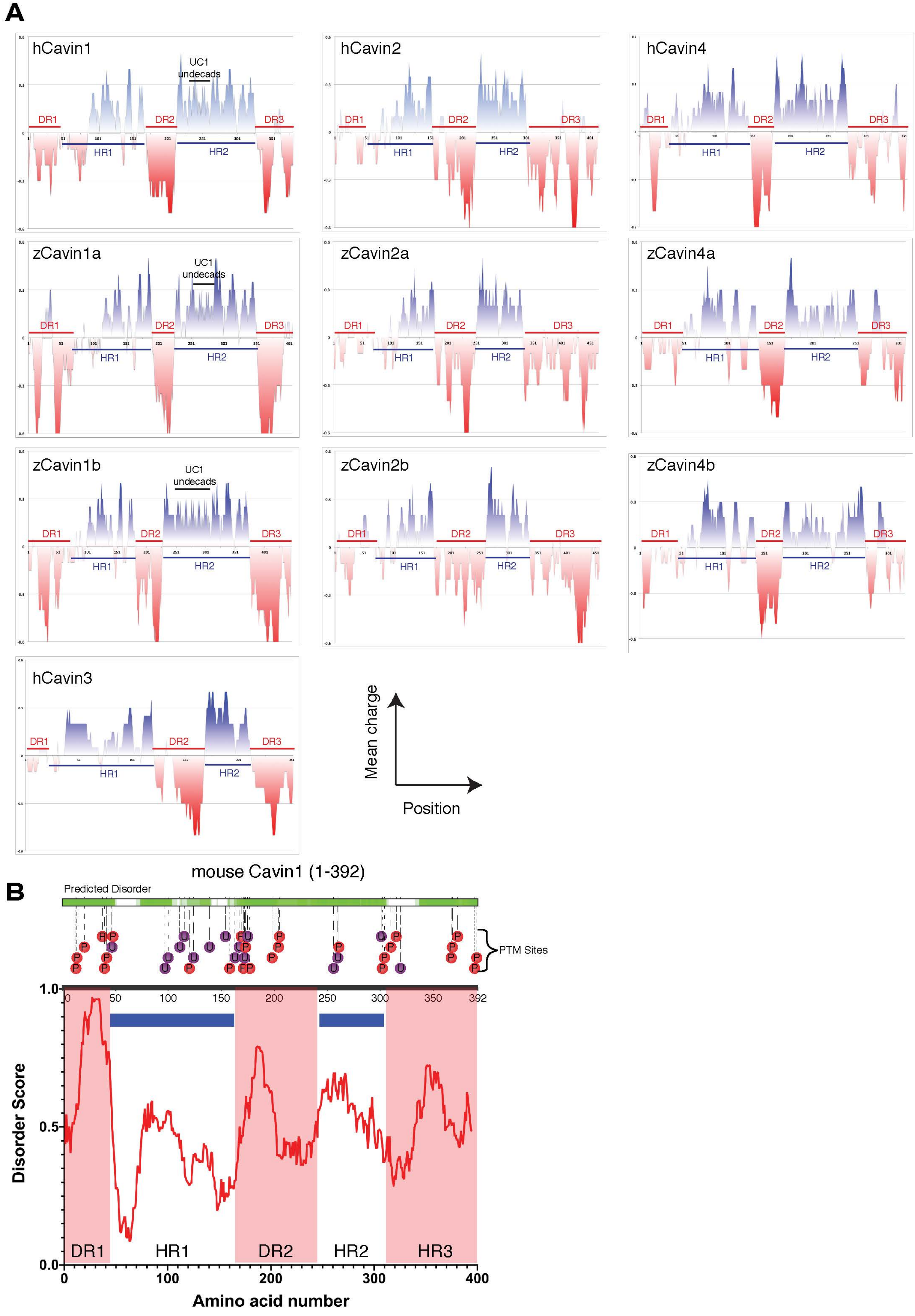

Supplementary Figure 1. Electrostatic charge distribution and sequence disorder in the Cavin family proteins.
(A) Protein charge plots of human (h) and zebrafish (z) cavin family proteins performed using the Emboss Server (http://www.bioinformatics.nl/cgi-bin/emboss/charge) (using standard input parameters and a window width of five amino acid residues). (B) The Cavin1 sequence was analysed using the D2P2 web server 1 for predicted regions of disorder, and also known sites of post-translational modifications.

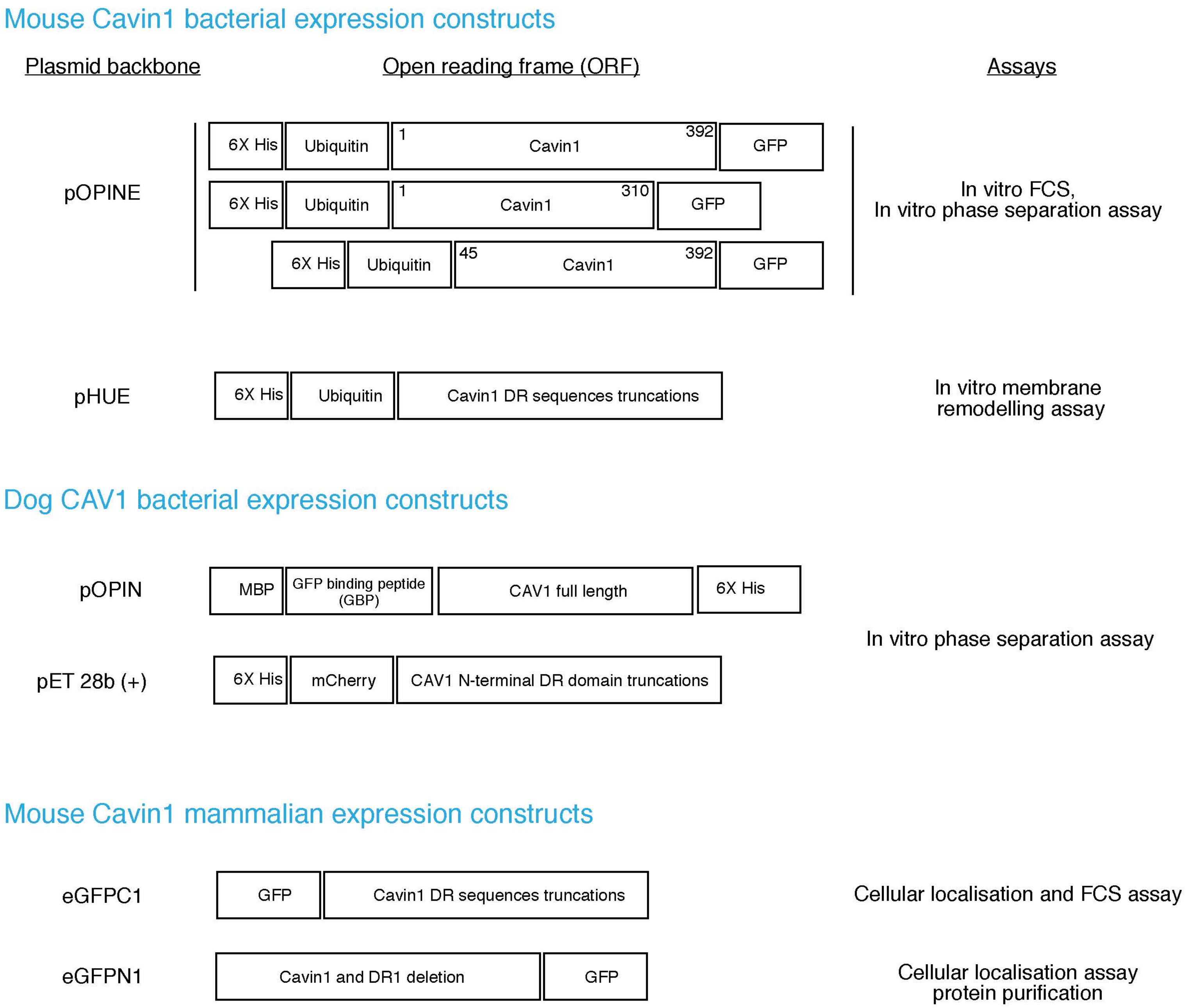

Supplementary Figure 2. Schematic representation of protein expression constructs used in this study.

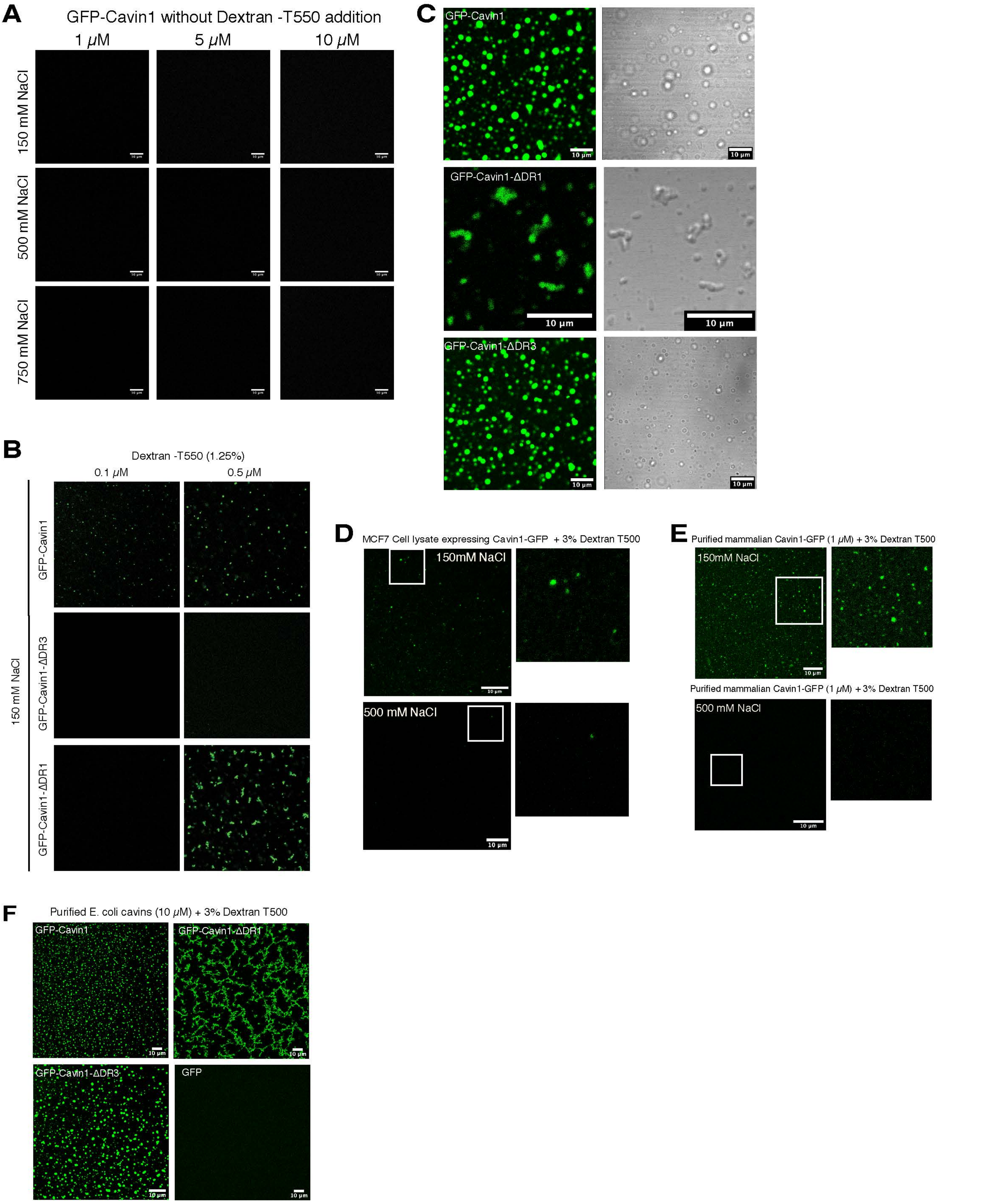

Supplementary Figure 3. LLPS behaviour of Cavin1 expressed and purified from bacteria and mammalian cells
(A) Liquid-liquid phase separation (LLPS) assay with bacterially expressed recombinant Ub- and GFP-tagged Cavin1, at different protein and salt concentrations but in the absence of dextran or other crowding agents. (B) At low concentrations, full length Cavin1 still forms liquid droplets, and Cavin1-ΔDR1 still forms coacervates. Cavin1-ΔDR3 is less prone to LLPS at low concentrations compared to the full-length protein. (C) LLPS assay performed with GFP tagged Cavin1-ΔDR1 by addition of 1.25% dextran T-500. Fluorescent GFP signal and adjacent bright filed image showing transparent drops unlike non-specific precipitates that are usually non-transparent and milky or brown in appearance. Scale bar – 10 μm; enlarged boxes are 10 μ m. (D) LLPS assay performed with Cavin1-GFP expressed and purified from mammalian HEK293 cells. (E) MCF7 cell lysates expressing Cavin1-GFP with the addition of 3% dextran T-500 in either 150 mM NaCl or 500 mM NaCl. Scale bar – 10 μm. (F) LLPS assay performed with purified E. coli cavins and GFP at higher dextran T-500 concentration (3%).

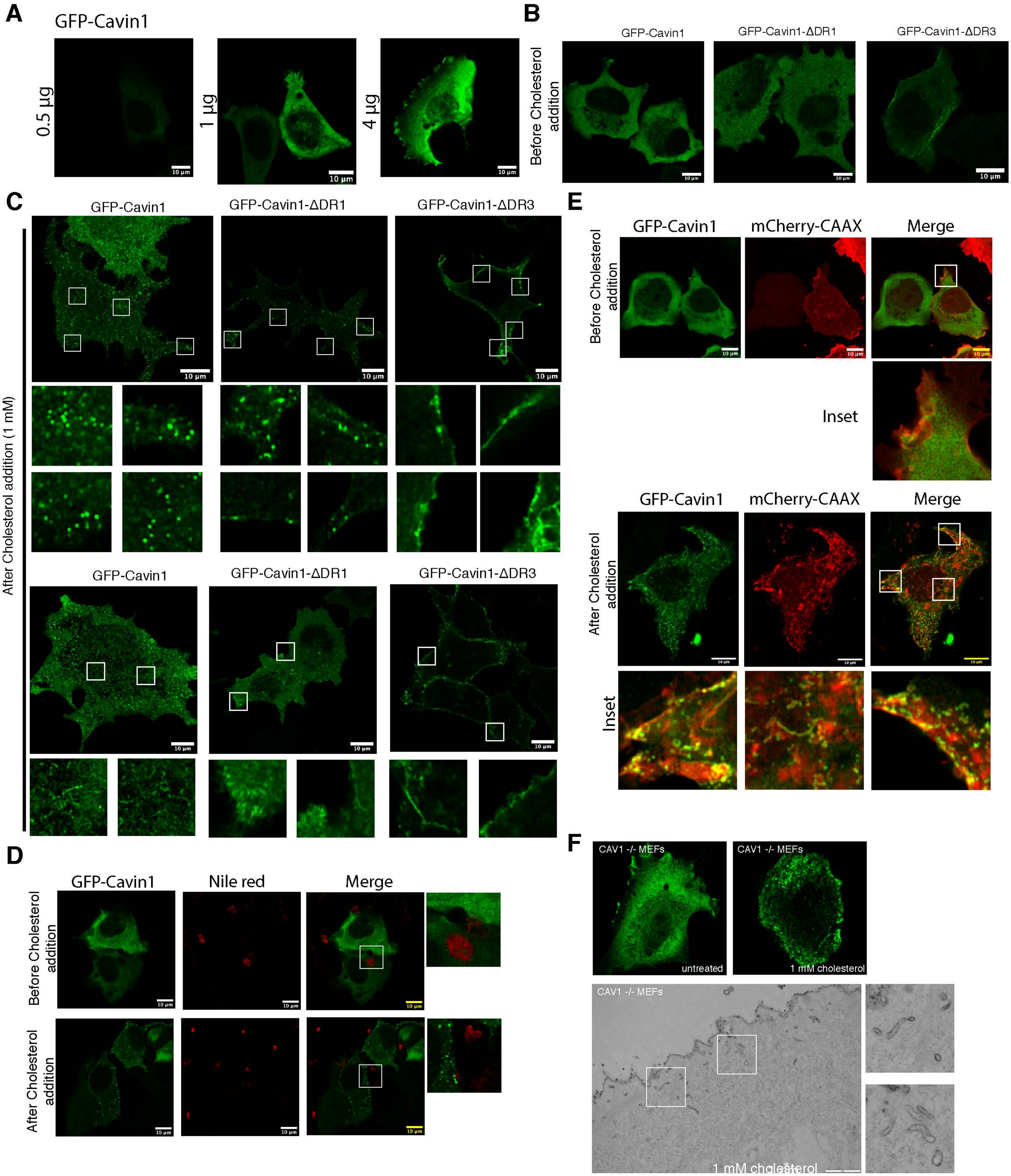

Supplementary Figure 4. Cavin1 undergoes LLPS and remodels cellular membranes devoid of CAV1.

(A) MCF7 cells expressing GFP-Cavin1 at varying amount of DNA showing cytosolic distribution. (B) MCF7 cells expressing GFP-Cavin1, Cavin1-ΔDR1 showing cytosolic distribution and Cavin1-ΔDR3 showing diffuse localisation (upper panel). (C) Addition of a water-soluble form of cholesterol (1 mM added, with effective available cholesterol concentration ~40 μM) to cells expressing GFP-Cavin1, Cavin1-ΔDR1 and Cavin1-ΔDR3 promotes liquid like droplet formation, membrane recruitment (upper panel) in some cells and tubulation in some cells for GFP-Cavin1 (lower panels). Scale bar – 10 μm; enlarged boxes are 5 μm. (D) MCF7 cells expressing GFP-Cavin1 with cholesterol addition formed GFP-Cavin1 condensates that did not stain with nile red suggesting these structures are not lipid droplets. Scale bar – 10 μm; enlarged boxes are 10 μm. (E) GFP-Cavin1 and mCherry-CAAX co-expression in MCF7 cells before cholesterol addition (upper panel) and after addition of cholesterol (lower panel) showing membrane patches and tubules partially co-localising with mCherry-CAAX. (F) CAV1-/- MEF cells expressing GFP-Cavin1 show cytosolic distribution and addition of 1 mM cholesterol causes membrane recruitment of GFP-Cavin1 (left panels) also observed by ruthenium red labelling of membrane surface by EM. Scale bar – 1 μm; enlarged boxes are 1 μm.

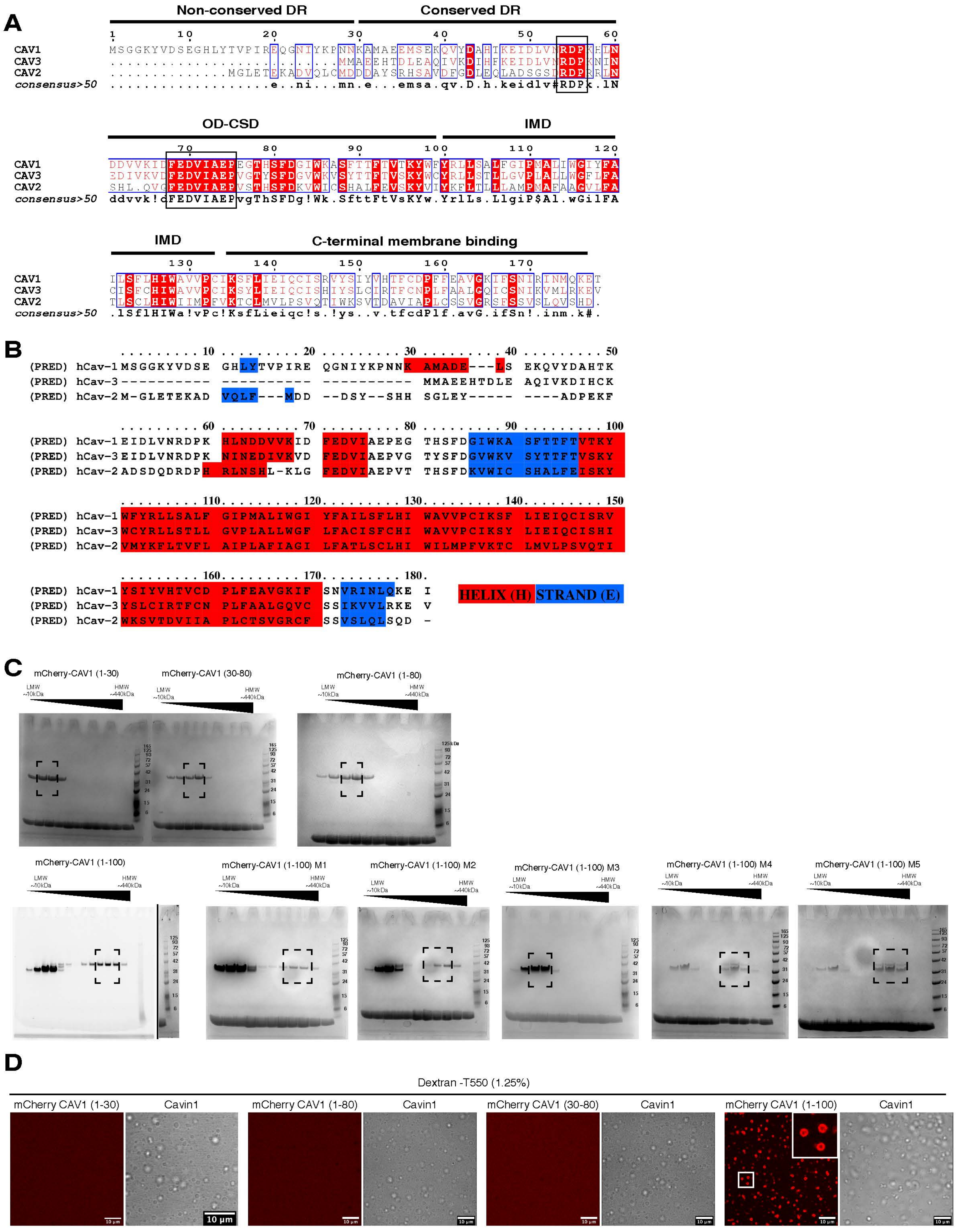

Supplementary Figure 5. Co-phase separation of CAV1 with Cavin1.

(A) Amino acid sequence alignment of dog caveolin sequences showing non-conserved and conserved fragments of N-terminal DR region, oligomerization and scaffolding domain (OD-CSD), intramembrane domain (IMD) and C-terminal membrane binding domain. (B) Alignment of human CAV1, CAV2 and CAV3 with secondary structure predictions performed using the Praline webserver (http://www.ibi.vu.nl/programs/pralinewww) 2. (C) In gel fluorescence images of gel filtration fractions for respective mCherry-tagged CAV1 mutants. Boxed areas indicate fractions pooled for further studies. (D) LLPS assay with mCherry CAV1 (1-30), (30-80), (1-80) and (1-100) and Cavin1. mCherry-CAV1 (1-100) is recruited to cavin1 droplets and undergo LLPS.

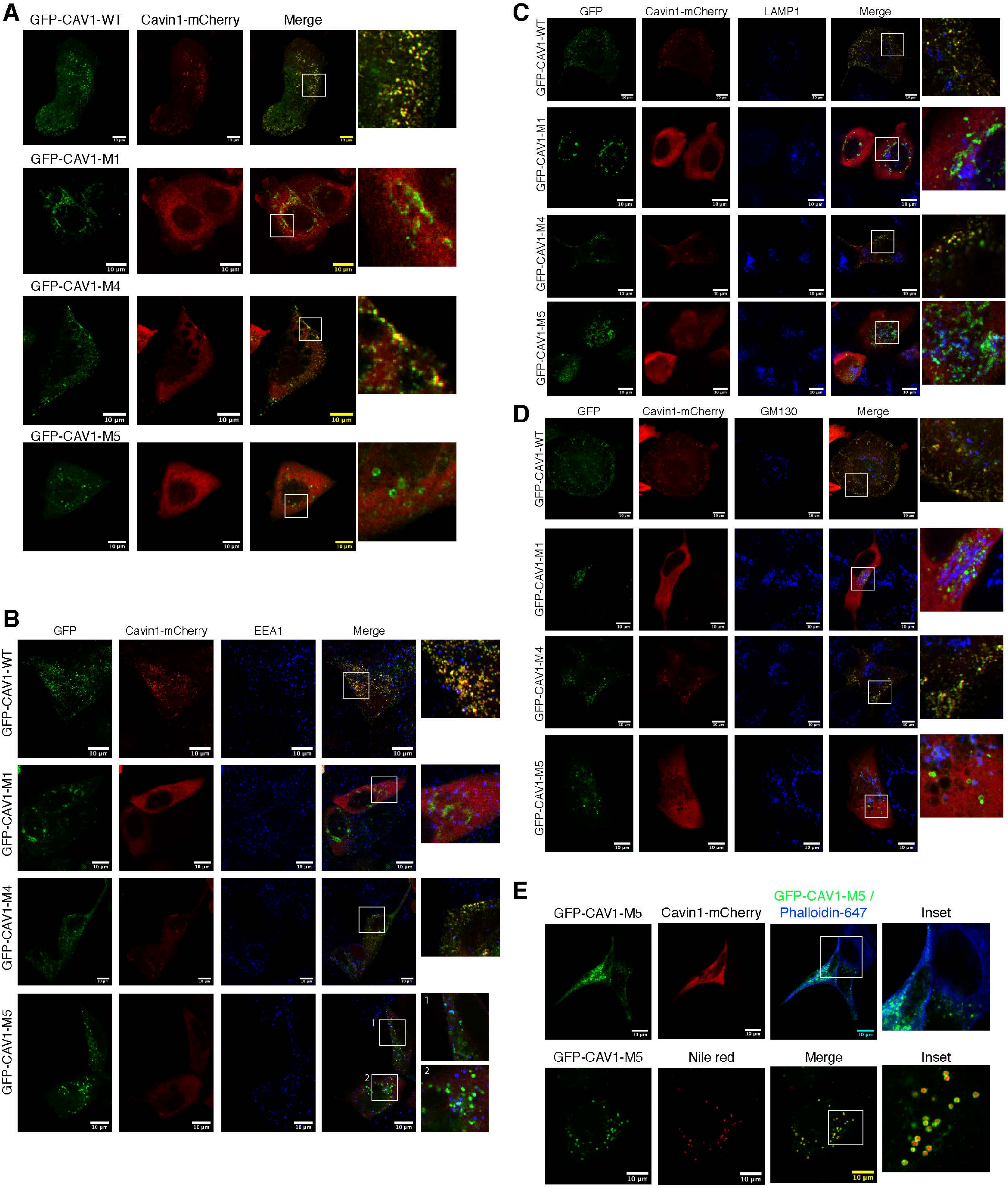

Supplementary Figure 6. Analysis of GFP-CAV1 mutants co-expressing Cavin1-mCherry in MCF7 cells.
GFP tagged CAV1 mutants (green) (Fig. 3) were co-expressed with Cavin1-mCherry in MCF7 cell line (A) and fixed cells were immunolabelled for early endosomes (EEA1) (B), lysosomes (LAMP1) (C), golgi membrane (GM130) (D), cellular actin (phalloidin) and nile red (lipid droplets) (E) Scale bar – 10 µm; enlarged boxes are 10 µm.

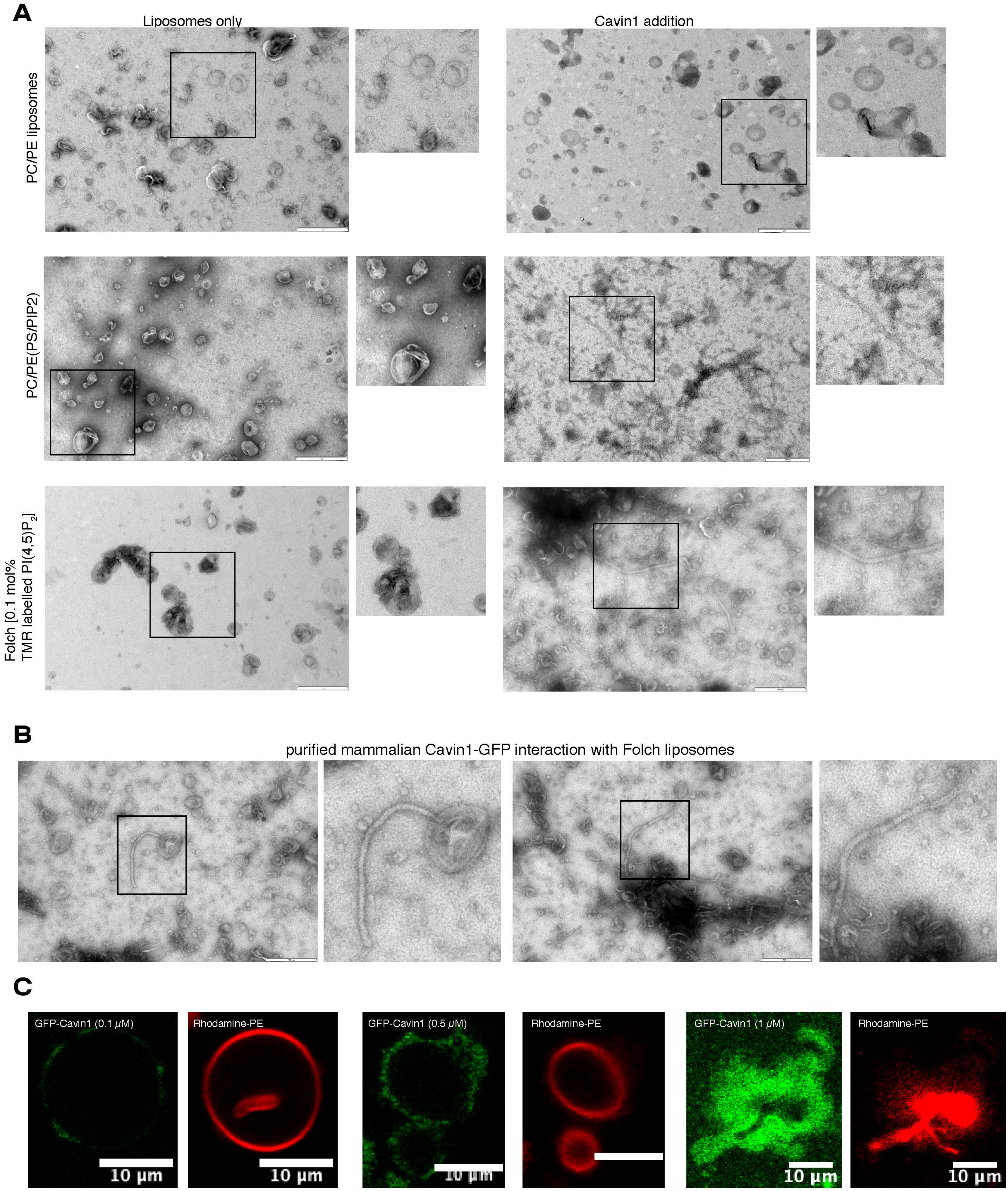

Supplementary Figure 7. Cavin1 membrane interactions in vitro

(A) In vitro membrane tubulation assay and negative stain electron microscopy was performed after mixing Cavin1 and liposomes consisting of Phosphatidylcholine (PC) and Phosphatidylethanolamine (PE) or PC/PE liposomes containing PI(4,5)P2 and Phosphatidylserine (PS) or Folch liposomes containing 0.1 mol% TMR labelled PI(4,5)P2 to replicate conditions in Figure 5. (B) In vitro membrane tubulation assay performed by mixing mammalian Cavin1-GFP with Folch liposomes, with membrane tubules highlighted in insets. Scale bar – 1 µm; enlarged boxes are 1.5 µm.. (C) Dose dependent GFP-Cavin1 interaction with GMVs containing rhodamine-PE.

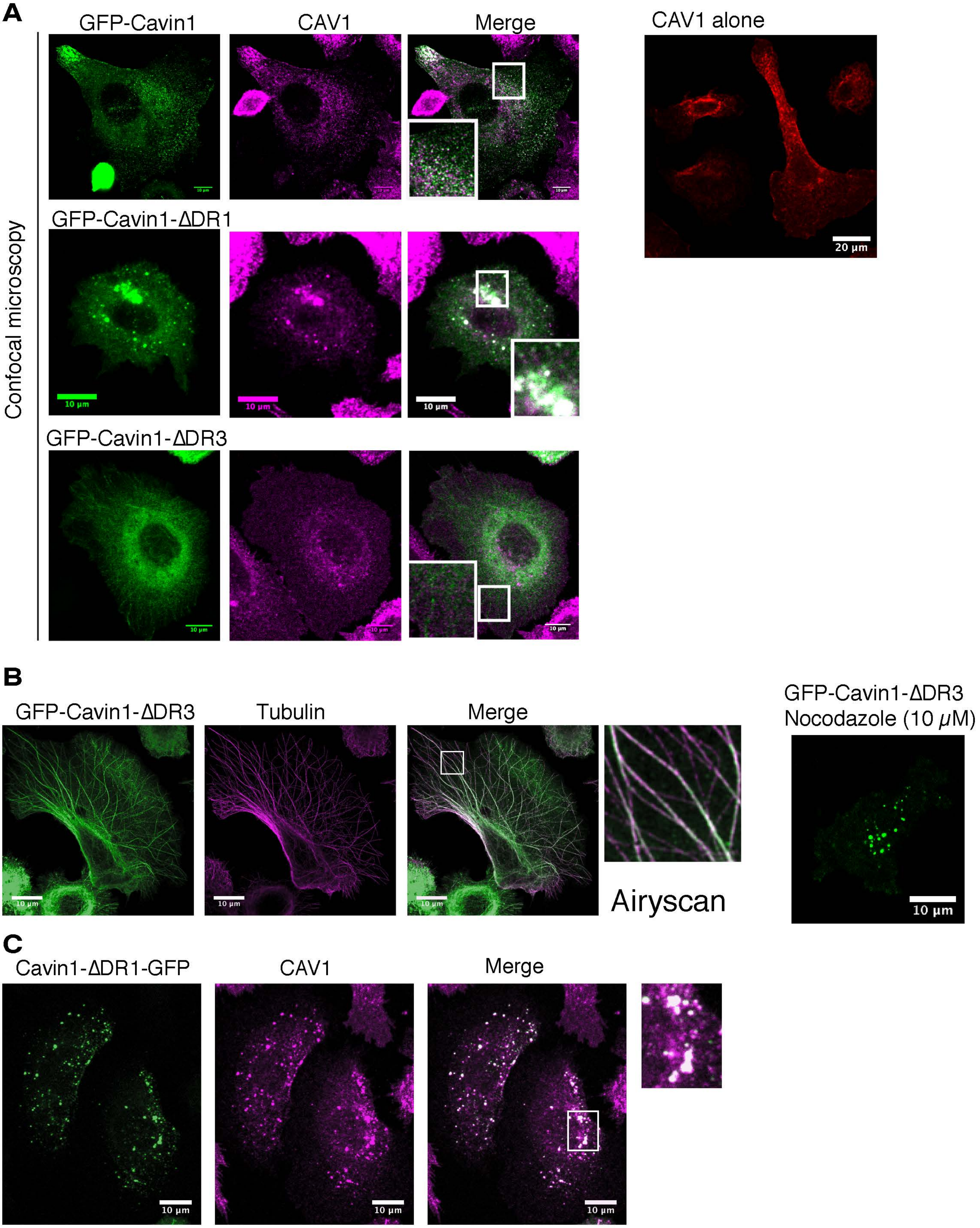

Supplementary Figure 8. Localisation of Cavin1 with truncated DR1 and DR3 domains.
(A) Confocal microscopy images of GFP-Cavin1, GFP-Cavin1-ΔDR1 and GFP-Cavin1-ΔDR3 immunolabelled with CAV1 (red) (B) GFP-Cavin1-ΔDR3 (green) associates with microtubules (red) in PC3 cells and disperses to the cytosol and forms liquid droplets after nocodazole treatment. Fluorescence images acquired with a Zeiss Airyscan2 microscope. (C) Cavin1-ΔDR1-GFP with a C-terminal GFP tag shows a similar intracellular accumulation with CAV1 in PC3 cells as the N-terminal GFP-tagged protein (Fig. 6A), suggesting that the GFP tag does not contribute to this phenotype. Scale bars – 10 µm; enlarged boxes are 10 µm.

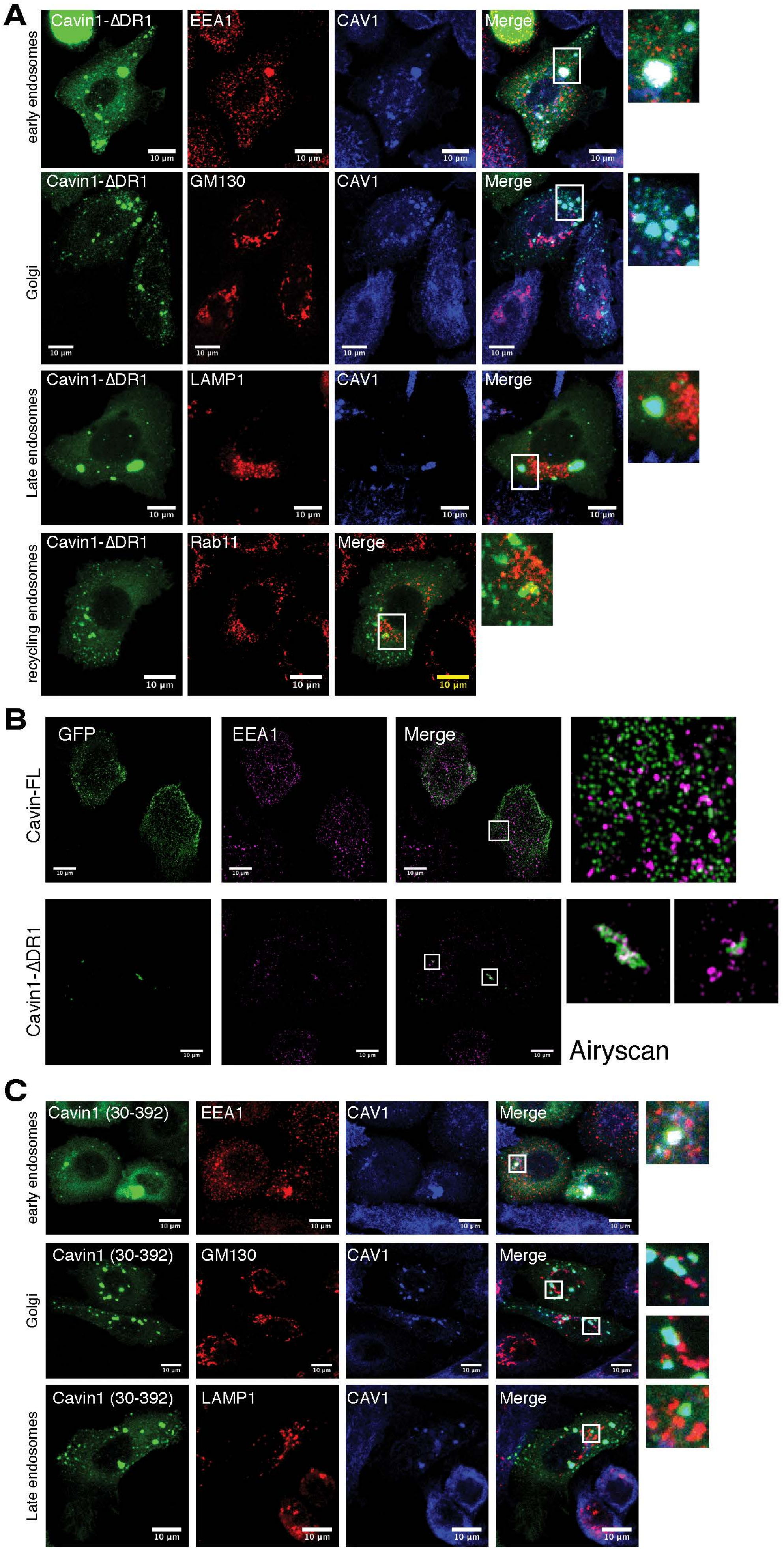

Supplementary Figure 9. Comparison of Cavin1 truncation mutants with endocytic markers.

(A) GFP-tagged Cavin1-ΔDR1 (green) was expressed in PC3 cells, and fixed cells were immunolabelled for CAV1 (blue) and different endocytic markers (red) including EEA1, GM130, LAMP1 and Rab11. Only EEA1 showed significant overlap with the internalised Cavin1-ΔDR1 and CAV1 positive structures. (B) High-resolution images of GFP-tagged Cavin1 and Cavin1-ΔDR1 (green) in PC3 cells compared with EEA1 (magenta) acquired with a Zeiss Airyscan2 microscope. (C) As for (A) but cells expressing GFP-tagged Cavin1(30-392). Cavin1(30-392) accumulates at intracellular structures with CAV1 and positive for EEA1 labelling similarly to Cavin1-ΔDR1 with the full deletion of the DR1 domain. Scale bar – 10 µm; enlarged boxes are 10 µm.

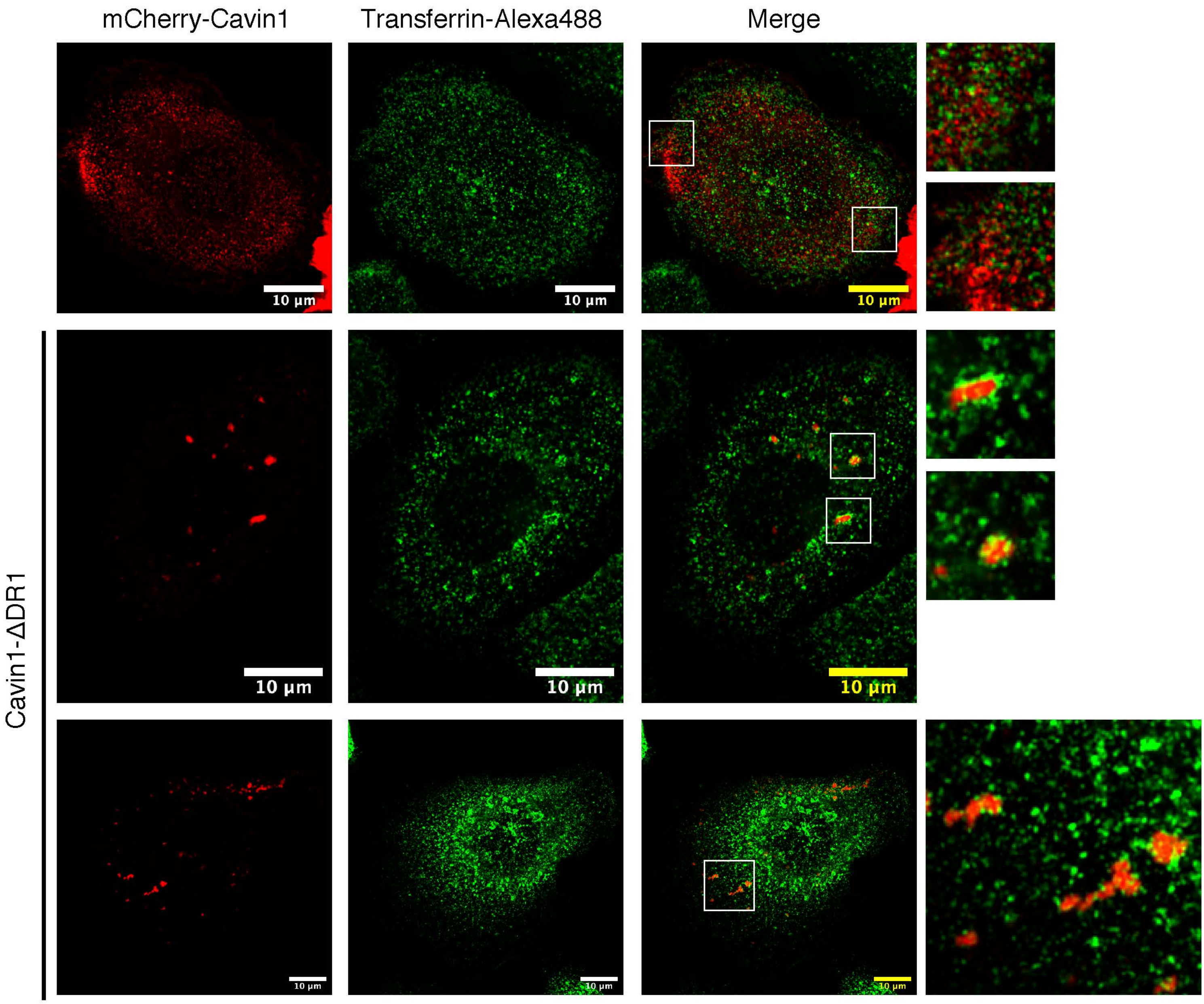

Supplementary Figure 10. Cavin1-ΔDR1 shows colocalisation with internalised transferrin.
Transferrin uptake assay was performed in PC3 cells expressing either mCherry-tagged Cavin1 or Cavin1-ΔDR1 (red) with transferrin Alexa-488 (green). Wild-type mCherry-Cavin1 showed no colocalization with endocytosed transferrin whereas mCherry-Cavin1-ΔDR1 formed large structures (red) with transferrin positive endosomes surrounding them.Scale bar – 10 μm; enlarged boxes are 10 μm.

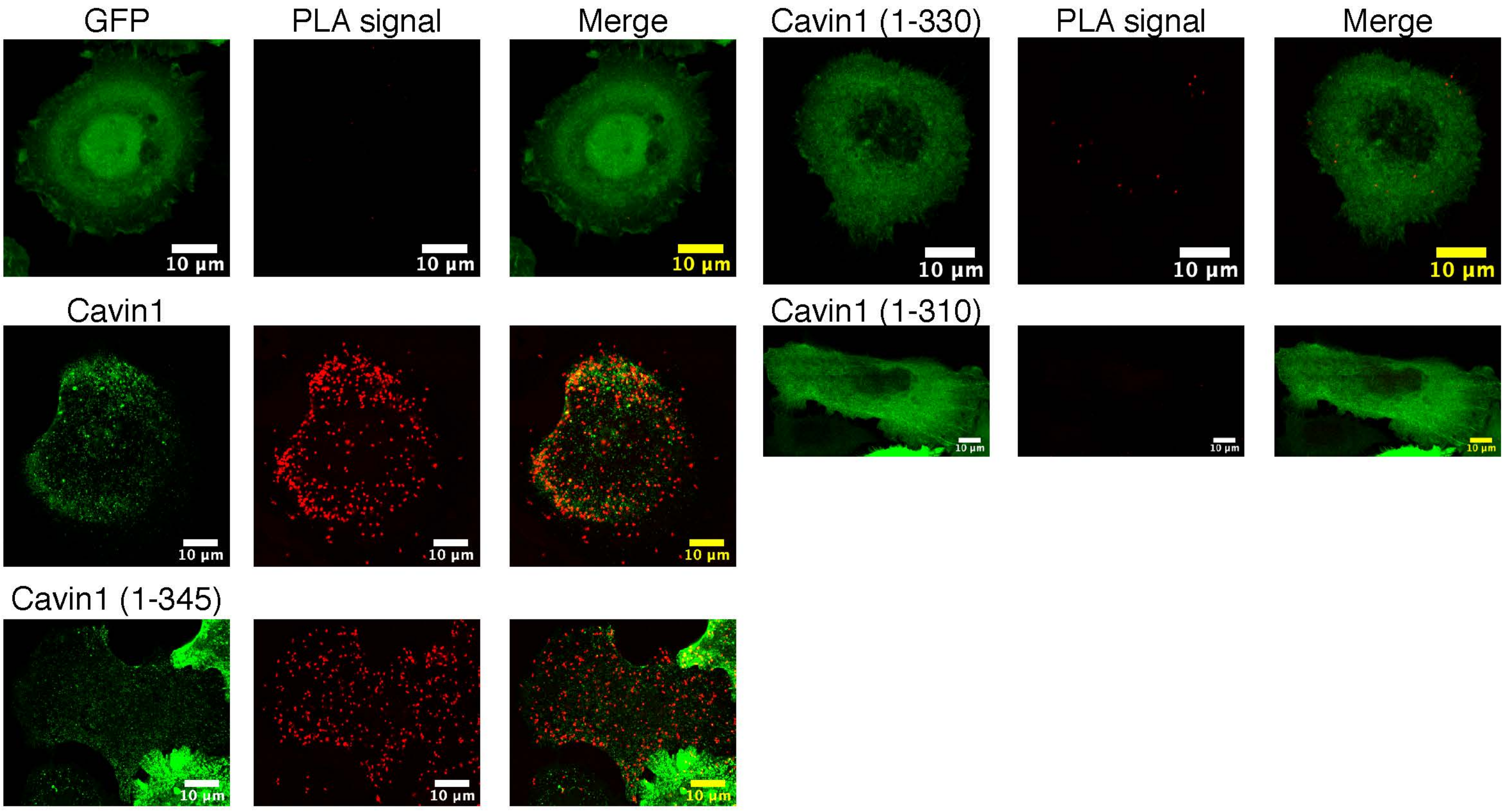

Supplementary Figure 11. PLA assay of Cavin1 interactions with CAV1
Representative images of proximity ligation assays of Cavin1 and CAV1 interactions, with GFP-tagged Cavin1 mutants in green and PLA signal in red. Scale bar – 10 µm. Related to Fig. 7C.

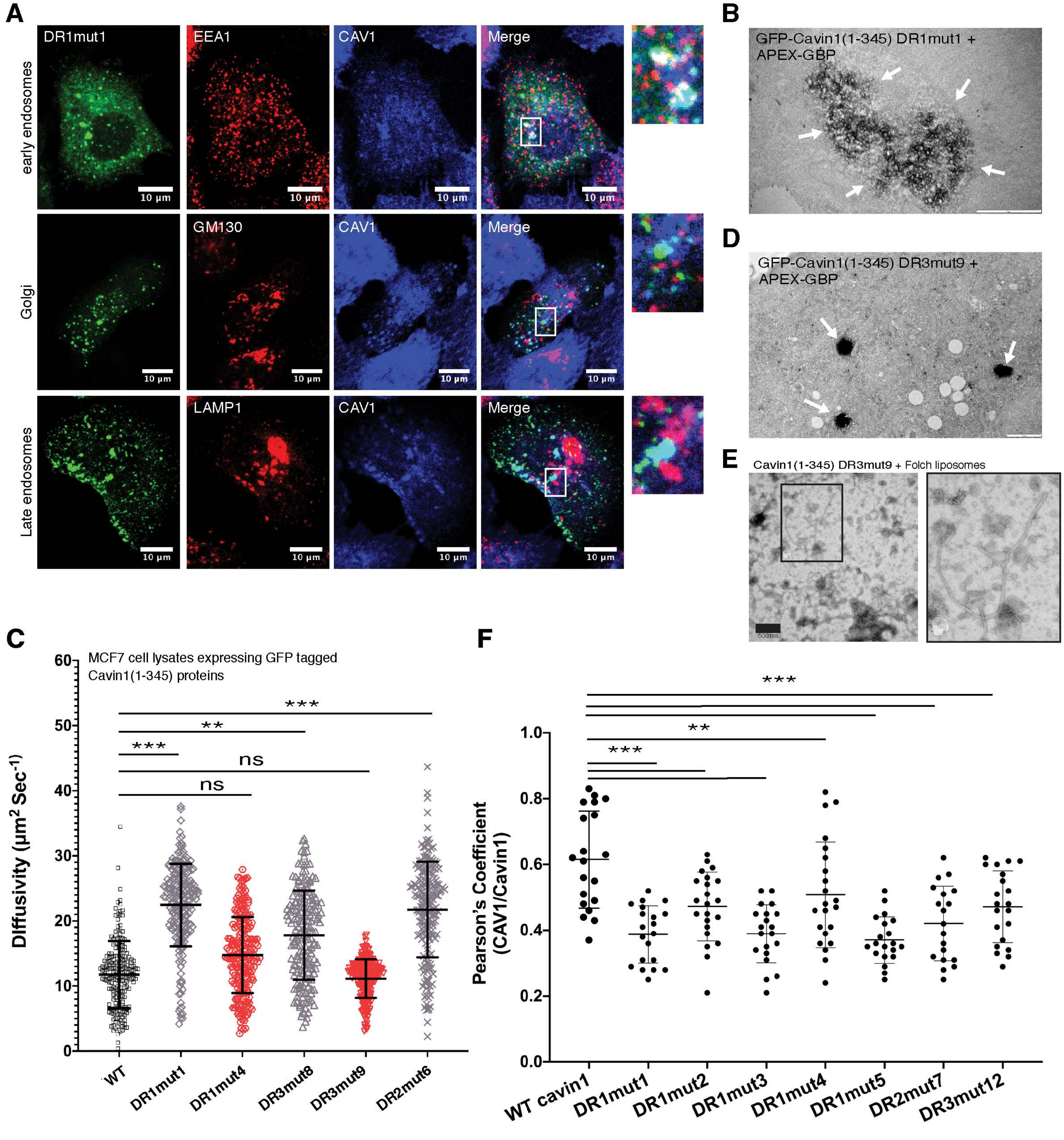

Supplementary Figure 12. Localisation and membrane remodelling by Cavin1(1-345) mutant proteins.
(A) GFP-tagged Cavin1(1-345) mutant DR1mut1 was expressed in PC3 cells, and fixed cells were immunolabelled for CAV1 (blue) and different endocytic markers (red) including EEA1, GM130, and LAMP1. Like the complete deletion of the residues 1-30 in the Cavin1 DR1 region (Fig. S9C) Cavin1(1-345) mutant DR1mut1 shows significant overlap with CAV1 and EEA1 positive internal structures. Scale bar – 10 µm; enlarged boxes are 5 µm. (B) APEX-GBP labelling of GFP-tagged Cavin1(1-345) mutant DR1mut1 shows accumulation and clustering with internal membrane vesicles (arrows). Scale bar – 1 µm. (C) The diffusion rate measured by FCS of selected GFP-tagged Cavin1(1-345) DR mutants in lysates after expression in MCF7 cells (lacking endogenous Cavins and Caveolins). n=184 over three independent experiments. Error bars indicate mean ± SD, **P<0.05, *** P<0.001, ns – not significant. (D) APEX-GBP labelling of GFP-tagged Cavin1(1-345) mutant DR3mut9 shows droplet localisation (arrows). Scale bar – 1 µm. (E) Purified Ub-tagged Cavin1(1-345) mutant DR3mut9 was mixed with unilamellar Folch liposomes (extruded to 400 nm diameter) and analysed by negative stain EM (1% uranyl acetate). This mutant is able to remodel and tubulate these synthetic membranes, although with a slightly larger diameter than wild-type Cavin or Cavin1(1-345) (Fig. 4D). Scale bar – 500 nm; enlarged box is 1 µm. (F) GFP-Cavin1 and various DR mutants of Cavin1 (1-345) were expressed in PC3 cell line and immunolabelled for CAV1 after fixation. The co-colocalization of GFP tagged cavin variants and CAV1 was quantified by Pearson's correlation coefficient. n=19 over two independent experiments. Error bars indicate mean ± SD. **P<0.05, *** P<0.001. Source data for Fig. S12C and S12F are provided as a Source Data file.

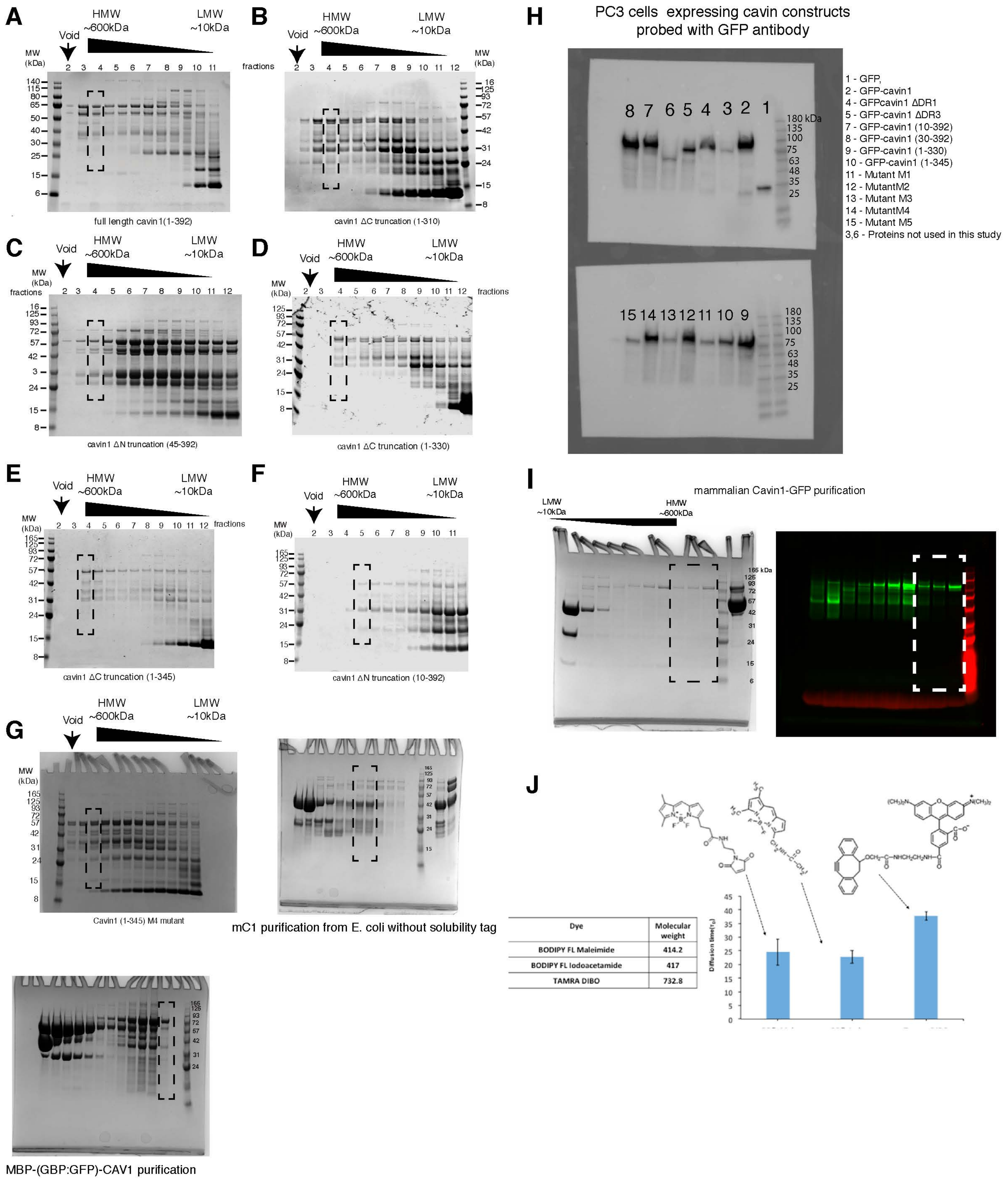

Supplementary Figure 13. (A to G) Gels showing purified recombinant Cavin1 proteins used in this study. (H) Western blot showing expression of GFP tagged mutants expressed in PC3 cell line probed with anti-GFP antibody. Boxed areas indicate fractions pooled for further studies. (I) SDS-PAGE and in gel fluorescence profile of Cavin1-GFP purified from HEK cells using GFP nanobody and subjected size exclusion chromatography on superose 6 (10/300) column. (J) The diffusion time measurements for three dyes performed before each FCS session. n=15 over two independent experiments. Data is shown as mean ± S.D.
