# Supplementary material for: Cavin1 intrinsically disordered domains are essential for fuzzy electrostatic interactions and caveola formation
Source: Nat Commun. 2021 Feb 10;12:931. doi: 10.1038/s41467-021-21035-4 (PMC7875971; doi:10.1038/s41467-021-21035-4)
Supplement: Supplementary file 3 — Description of Additional Supplementary Files [file 41467_2021_21035_MOESM3_ESM.pdf]

Description of Additional Supplementary Files

File Name: Supplementary Movie 1
Description: (related to Fig. 4B). Cryoelectron tomography (CryoET) of Cavin1-coated membrane tubules. The movies shows a series of images panning through the three-dimensional tomographic volume. Striated protein densities are observed coating the relatively heterogeneous membrane tubules.

File Name: Supplementary Movie 2
Description: (related to Fig. 6B). GFP-tagged Cavin1-ΔDR3 was expressed in PC3 cells and photobleaching was performed on a small region along microtubules coated with GFP tagged mutant protein. Images were acquired one frame per second.

File Name: Supplementary Movie 3
Description: (related to Fig. 6C). GFP-tagged Cavin1- ΔDR3 was expressed in PC3 cells and treated with nocodazole (10 µM). Photobleaching was performed on a small region containing liquid droplets of mutant protein and images were acquired one frame per second.

File Name: Supplementary Movie 4
Description: (related to Fig. 6F). GFP-tagged Cavin1 and Rab5a-mCherry were co-expressed in PC3 cells and images were acquired one frame per four seconds.

File Name: Supplementary Movie 5
Description: (related to Fig. 6F). GFP-tagged Cavin1- ΔDR1 and Rab5a-mCherry were co-expressed in PC3 cells and images were acquired one frame per four seconds.

File Name: Supplementary Movie 6
Description: (Related to Fig. 8E). GFP-Cavin1 DR3mut9 mutant expressed in PC3 cell line and photobleaching was performed on protein droplets dispersed in cytosol. Images were acquired one frame per two seconds.
