# Supplementary material for: Cavin1 intrinsically disordered domains are essential for fuzzy electrostatic interactions and caveola formation
Source: Nat Commun. 2021 Feb 10;12:931. doi: 10.1038/s41467-021-21035-4 (PMC7875971; doi:10.1038/s41467-021-21035-4)
Supplement: Supplementary file 10 — Reporting Summary [file 41467_2021_21035_MOESM10_ESM.pdf]

nature research

Corresponding author(s): Robert G. Parton and Brett M. Collins

Last updated by author(s): May 27, 2020

Reporting Summary

Nature Research wishes to improve the reproducibility of the work that we publish. This form provides structure for consistency and transparency in reporting. For further information on Nature Research policies, see our Editorial Policies and the Editorial Policy Checklist.

Statistics

For all statistical analyses, confirm that the following items are present in the figure legend, table legend, main text, or Methods section.

n/a	Confirmed	
☐	☒	The exact sample size (*n*) for each experimental group/condition, given as a discrete number and unit of measurement
☐	☒	A statement on whether measurements were taken from distinct samples or whether the same sample was measured repeatedly
☐	☒	The statistical test(s) used AND whether they are one- or two-sided<br>*Only common tests should be described solely by name; describe more complex techniques in the Methods section.*
☒	☐	A description of all covariates tested
☒	☐	A description of any assumptions or corrections, such as tests of normality and adjustment for multiple comparisons
☐	☒	A full description of the statistical parameters including central tendency (e.g. means) or other basic estimates (e.g. regression coefficient) AND variation (e.g. standard deviation) or associated estimates of uncertainty (e.g. confidence intervals)
☐	☒	For null hypothesis testing, the test statistic (e.g. F, t, r) with confidence intervals, effect sizes, degrees of freedom and P value noted<br>*Give P values as exact values whenever suitable.*
☒	☐	For Bayesian analysis, information on the choice of priors and Markov chain Monte Carlo settings
☒	☐	For hierarchical and complex designs, identification of the appropriate level for tests and full reporting of outcomes
☐	☒	Estimates of effect sizes (e.g. Cohen's d, Pearson's r), indicating how they were calculated

Our web collection on statistics for biologists contains articles on many of the points above.

Software and code

Policy information about availability of computer code

Data collection	iTEM (Version 5.2)<br>Tomography (Thermo Fisher Scientific-FEI, NL) (Version 4)
Data analysis	Graph Pad<br>Prism (Version 8)<br>COOT (Version 0.8.2)<br>PHENIX (Version 1.14)<br>PYMOL (Version 2.3.1)<br>Zeiss software (ZEN 2.3)<br>ImageJ (1.50g)<br>IMOD (https://bio3d.colorado.edu/imod/ version 4.9)

For manuscripts utilizing custom algorithms or software that are central to the research but not yet described in published literature, software must be made available to editors and reviewers. We strongly encourage code deposition in a community repository (e.g. GitHub). See the Nature Research guidelines for submitting code & software for further information.

Data

Policy information about availability of data

All manuscripts must include a data availability statement. This statement should provide the following information, where applicable:

- Accession codes, unique identifiers, or web links for publicly available datasets
- A list of figures that have associated raw data
- A description of any restrictions on data availability

Source data for Figs. 1,2, 6, and 7 are provided in Table S1. The data that support the findings of this study are available from the corresponding author on request. For Figure 9A the model of Cavin1 incorporates PDB coordinates 4QKV

Field-specific reporting

Please select the one below that is the best fit for your research. If you are not sure, read the appropriate sections before making your selection.

- [x] Life sciences
- [ ] Behavioural & social sciences
- [ ] Ecological, evolutionary & environmental sciences

For a reference copy of the document with all sections, see nature.com/documents/nr-reporting-summary-flat.pdf

Life sciences study design

All studies must disclose on these points even when the disclosure is negative.

Sample size	Sample sizes for different experiments were chosen based on prior knowledge for obtaining significant p-values from the type of experiment performed. For examples see Tillu et al. (Cell Reports, 2018) and Tillu et al. (Mol. Biol. Cell, 2015)<br>The study did not involve animal experiments and/or human research participants.
Data exclusions	No experimental data sets determined were excluded. No "outlier" analyses and exclusions were done.
Replication	All results shown as representative data have been reproduced (please see Methods and Figure legends for details of how many independent experiments yielding similar results were done).
Randomization	Cells were randomly selected for imaging.
Blinding	No blinding was performed since the same person performed and analyzed the data.

Reporting for specific materials, systems and methods

We require information from authors about some types of materials, experimental systems and methods used in many studies. Here, indicate whether each material, system or method listed is relevant to your study. If you are not sure if a list item applies to your research, read the appropriate section before selecting a response.

Materials & experimental systems

n/a	Involved in the study	
☐	☒	Antibodies
☐	☒	Eukaryotic cell lines
☒	☐	Palaeontology and archaeology
☒	☐	Animals and other organisms
☒	☐	Human research participants
☒	☐	Clinical data
☒	☐	Dual use research of concern

Methods

n/a	Involved in the study	
☒	☐	ChIP-seq
☒	☐	Flow cytometry
☒	☐	MRI-based neuroimaging

Antibodies

Antibodies used	Antibodies used were as follows, rabbit polyclonal anti-Caveolin1 (BD Transduction Laboratories, Cat. No. 610060), mouse monoclonal anti-GFP (Roche Diagnostics Cat. No. 11814460001), Donkey anti-Rabbit IgG (H+L) Secondary Antibody Alexa Fluor® 555 conjugate (Thremo Fisher Scientific, Cat No. A31572). Mouse monoclonal anti-tubulin (Anti-alpha Tubulin antibody [DM1A] - Abcam (ab7291)).
Validation	Validation from supplier websites:<br>anti-Caveolin1 (BD Transduction Laboratories, Cat. No. 610060)

QC Testing: Human. Tested in Development: Mouse, Rat, Dog, Chicken. Recommended for Western blot.

anti-GFP (Roche Diagnostics Cat. No. 11814460001)
Validated for immunoprecipitation, Western blotting and immunostaining

anti-tubulin (Anti-alpha Tubulin antibody [DM1A] - Abcam (ab7291)
Tested applications Suitable for: Flow Cyt, ICC/IF, IP, IHC-Fr, IHC-P, Electron Microscopy, WB.

Eukaryotic cell lines

Policy information about cell lines

Cell line source(s)	PC3 and MCF7 cells were originally sourced from the ATCC.
Authentication	Authentication was from the ATCC. We have not independently authenticated the cell lines.
Mycoplasma contamination	Cell lines were tested fortnightly for mycoplasma contamination and were confirmed negative.
Commonly misidentified lines (See ICLAC register)	No commonly misidentified cell lines were used in this study
