# Supplementary material for: Cavin1 intrinsically disordered domains are essential for fuzzy electrostatic interactions and caveola formation
Source: Nat Commun. 2021 Feb 10;12:931. doi: 10.1038/s41467-021-21035-4 (PMC7875971; doi:10.1038/s41467-021-21035-4)
Supplement: Supplementary file 2 — Peer Review File [file 41467_2021_21035_MOESM2_ESM.pdf]

Editorial Note: This manuscript has been previously reviewed at another journal that is not operating a transparent peer review scheme. This document only contains reviewer comments and rebuttal letters for versions considered at Nature Communications.

REVIEWERS' COMMENTS

Reviewer #1 (Remarks to the Author):

In the revised manuscript, the authors have performed additional control experiments and tempered some of the conclusions, which has further strengthened the data and improved the manuscript. My previous comments have been adequately addressed and the authors have addressed all comments raised by the other reviewers by new experiments, rewriting or clarifications. I believe that this manuscript significantly contributes to our understanding of mechanisms for protein assembly at the membrane interface. In particular, this manuscript provides a novel model for assembly of the cavin-proteins and biogenesis of the caveolae coat which has eluded scientist for decades.
